# Signal Amplification Gains of Compressive Sampling for Photocurrent Response Mapping of Optoelectronic Devices

**DOI:** 10.3390/s19132870

**Published:** 2019-06-28

**Authors:** George Koutsourakis, James C. Blakesley, Fernando A. Castro

**Affiliations:** 1 National Physical Laboratory (NPL), Hampton Road, Teddington, Middlesex TW11 0LW, UK; 2 Advanced Technology Institute, University of Surrey, Guildford, Surrey GU2 7XH, UK

**Keywords:** non-destructive testing, current mapping, digital micromirror device, compressed sensing

## Abstract

Spatial characterisation methods for photodetectors and other optoelectronic devices are necessary for determining local performance, as well as detecting local defects and the non-uniformities of devices. Light beam induced current measurements provide local performance information about devices at their actual operating conditions. Compressed sensing current mapping offers additional specific advantages, such as high speed without the use of complicated experimental layouts or lock-in amplifiers. In this work, the signal amplification advantages of compressed sensing current mapping are presented. It is demonstrated that the sparsity of the patterns used for compressive sampling can be controlled to achieve significant signal amplification of at least two orders of magnitude, while maintaining or increasing the accuracy of measurements. Accurate measurements can be acquired even when a point-by-point scan yields high noise levels, which distort the accuracy of measurements. Pixel-by-pixel comparisons of photocurrent maps are realised using different sensing matrices and reconstruction algorithms for different samples. The results additionally demonstrate that such an optical system would be ideal for investigating compressed sensing procedures for other optical measurement applications, where experimental noise is included.

## 1. Introduction

The non-uniformities of material structure and local defects can have an influence on the overall performance of optoelectronic devices, such as solar cells and photodiodes. Therefore, it is important to develop methods that provide spatially resolved information on the defects and inhomogeneities of such semiconductor devices. Light/laser beam induced current (LBIC) methods have been established for the spatial characterisation of solar cells [1], photodiodes [2,3], and other sensors and *p*–*n* junction devices [4,5]. For the realisation of current mapping, a light beam scans the device being tested, and the induced current is measured for every point. A variety of different system approaches have been proposed, making the LBIC measurement systems able to deliver spatial maps of electrical properties [6], local reflectivity [7], performance parameters [8], and material properties of optoelectronic devices. Recent implementations utilise multiple laser wavelengths that enable measurements on a larger range of samples and for different energy ranges [1,9].

Although useful and sometimes necessary, photoresponse mapping measurements are usually time-consuming, since a small spot size has to scan the entire active area of the device for a total area current map, which means the smaller the spot size, the lengthier the measurements. Focusing the laser beam on a small spot often requires elaborate optical elements and accurate alignment. A very frequent solution is to use a microscope objective lens to achieve a spot size of several micrometres [10]. The point-by-point scan is realised by an *x*–*y* stage, which means that there is always a time delay from moving from one point to the next. Continuous acquisition methods have been reported in order to accelerate this process [11]; nevertheless, this can result in small distortions of the current. The alternative option to maximise scan speed is to use piezo-electric mirror systems to guide the beam on the sample [12,13]. Spot sizes of several micrometres also mean very weak signals. For this reason, lock-in techniques for accurate current readings were introduced, even in very early systems [14], and have been used in almost every LBIC system implementation ever since. Combining all of the above features into one system is not trivial, and LBIC systems can become very complicated to realise.

The first attempt to utilise digital light processing (DLP) for current mapping was by a fast tomographic current mapping method for photovoltaic (PV), based on a digital micromirror device (DMD) for implementing the scan [15]. DLP devices utilise a DMD to create light projections [16]. Photocathode quantum efficiency mapping using a digital micromirror device has been reported, where the DMD implements the laser raster scan [17]. A DLP projector has been utilised for low-resolution spatial uniformity characterisation of solar cells [18]. A DMD-based system has also been reported for fast spectral response measurements of PV devices [19], where additional frequency modulation for each wavelength band has been reported, in order to accelerate measurements [20]. High-frequency light modulation of more than 40 GHz has also been introduced recently, with an Si light emitter embedded in a *p*-channel, metal oxide, semiconductor field effect transistor (PMOSFET) structure [21].

Using a DMD to apply compressed sensing (CS) current mapping of PV devices has been demonstrated in recent work [22,23,24]. CS current mapping has also been demonstrated by utilising an LCD (liquid crystal display) monitor to project the necessary patterns for compressive sampling [25]. The CS current mapping method is based on the CS sampling theory [26,27]. According to this theory, it is possible to reconstruct a signal from highly incomplete or inaccurate information. Compression of signals is something very common in everyday life. For instance, in JPEG image compression, most of the signal information is thrown away at the transform compression stage. Only the necessary elements for describing the image in the transform domain are kept (*K* elements). The image is reconstructed using these very few *K* elements, which provide a sparse representation of the image. The aim of CS imaging is to directly acquire the *K* coefficients necessary for an almost exact reconstruction of a signal. This is achieved by only acquiring *M* < *N* measurements for capturing an *N* pixel image, where *K* < *M*. There are a large number of compressive sampling applications, such as CS Magnetic resonance imaging (MRI) [28], the single-pixel camera [29], CS radar imaging [30], CS confocal microscopy [31], and many more. 

In previous work, we have presented the CS current mapping methodology and a design for a CS current mapping measurement system for solar cells [22,23]. In this work, the signal amplification aspects of the sampling process and technical approaches for optimised sampling are presented. Although the performance of different CS aspects (algorithms, transforms, and matrices) can be investigated by simulations, aberrations of compressive sampling due to instrumentation and optics only show in experimental investigations, such as the one presented in this work. The significant signal amplification gains of CS current mapping for optoelectronic devices are demonstrated and discussed. The optimum ways to achieve such amplification for the measured signal and the impact of sensing matrix sparsity (defined later on) on the accuracy of measurements are investigated for the first time for such an application. Three types of devices are used to illustrate that the choice of sensing matrix sparsity is dependent of sample and measurement instrumentation. The robustness of CS current mapping against long-term measurement noise is studied, and a pixel-by-pixel comparison of compressive and point-by-point sampling for current mapping is realised. Different measurement settings and samples are tested using the DMD optical system. This comparison aids in determining the most suitable occasions in which each sampling method should be realised, and presents a realistic performance evaluation of compressive sampling for this specific application. In addition, it is demonstrated that this optical setup is ideal for realistic experimental comparisons of reconstruction algorithms for optical measurement applications of compressed sensing.

## 2. Methodology

### 2.1. Experimental Layout

**Figure 1:**
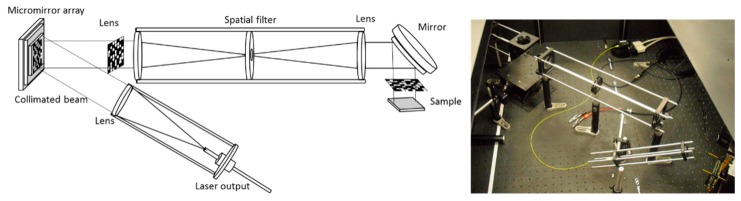
On the left, schematic of the optical system used for compressed sensing (CS) current mapping. On the right, a photo of the system.

**Figure 2:**
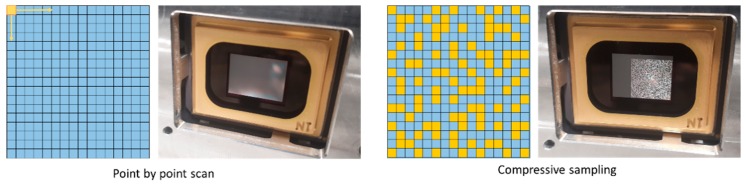
Schematics of the two different sampling modes used, and photos of how each is implemented on the digital micromirror device (DMD): on the left, point-by-point sampling; on the right, compressive sampling.

The optical current mapping system used in this work is based on a DMD kit and is presented in Figure 1. A single mode fibre-coupled laser source of 40 mW at a 637 nm wavelength is used. The light output of the fibre is collimated such that the beam overfills the DMD micro-mirror area. The DMD is a V-7000 module, consisting of a 1024 × 768 pixel micromirror array, each micromirror having a pixel size of 13.7 × 13.7 μm. A spatial filter is used to reject the diffracted and non-collimated components of the beam. Finally, a mirror is used for guiding the beam onto the sample, which is placed horizontally on a *z*-stage platform. A National Instruments PXIe-4139 source measure unit (SMU) is used for measuring the current for both cases of sampling (raster scanning and patterns). The sample is placed at the focal plane of the last lens, so that the scanning spot or the patterns are projected onto the sample. In order to apply a compressive or a point-by-point scan, a number of micromirrors are grouped together to form one pixel, and the number of grouped micromirrors depends on the selected optical resolution. The spot shape is square. The sampling methods are presented in Figure 2. As can be seen in the picture of the DMD on the right of Figure 2, not all of the active area of the DMD (1024 × 768 pixels) is used. A square 700 × 700 pixel area is used to project the patterns, in order to create a square projection. Groups of 7 × 7 micromirrors are binned together, creating projections of 100 × 100 pixels. This results in a 100 × 100 resolution of the final current maps. The sampling rate that can be achieved is 30 points or patterns per second and this sampling rate is used for all silicon samples of this work. For the organic device measured, a slower sampling rate was selected (5 samples/s), due to the slower response of the specific organic photovoltaic device [32].

### 2.2. Compressed Sensing Current Mapping

For the application of compressive sampling, a series of binary patterns are projected onto the sample’s area to be measured, and the photocurrent response of the sample is measured for each pattern. The patterns are generated by the DMD, assigning pixels (binned groups of micromirrors) as either “on” or “off”, illuminating or shading different points of the sample, as can be seen in Figure 2. Similar to JPEG image compression, the sequence of patterns measures and compresses the necessary information, in order to successfully reconstruct the photocurrent response map. This is a standard procedure for optical CS imaging systems [33] that is analytically described for CS current mapping of photovoltaic (PV) devices in [34]. Compared to a point-by-point scan, fewer measurements are required in order to produce a current map of a sample.

In summary, for the application of CS current mapping, a series of binary patterns $\Phi \;=\;\{\varphi_{m}{\}}_{m=1}^{M}$ are projected onto the sample, in order to acquire a compressed representation of the signal **x**, which has *N* elements, using *M* < *N* linear measurements. Each row of the sensing matrix Φ is a binary pattern expressed as a vector, which makes Φ an *N* × *M* matrix. The current response of the PV device is measured for each pattern, populating the measurement vector **y**. An underdetermined problem **x**: y = Φx is created, since **y** has fewer elements than x. Random binary matrices are used in this work to produce the sensing matrix, as it has been shown that they possess the necessary properties needed for compressive sampling [35]. The discrete cosine transform (DCT) is applied as a basis to provide the sparse representation of the signal. Two different algorithms are used in this work for solving this underdetermined problem and reconstructing the current map. The first is the ℓ1 norm minimisation basis pursuit algorithm, included in the ℓ1 magic toolbox in MatLab developed by Candès and Romberg [36]. A second algorithm used is the orthogonal matching pursuit (OMP) algorithm [37]. Using one of the above algorithms, the underdetermined problem is solved, and the current map is reconstructed. 

Although it is not within the scope of this work to investigate different reconstruction algorithms, the right choice of algorithm can be crucial for the successful reconstruction of the final current map. Nevertheless, given a specific algorithm, the choice of sensing matrix sparsity does not significantly affect the reconstructed image, as will be demonstrated in this work. This is shown by acquiring similar current mapping results when using different sensing matrices, for each of the two algorithms. In addition, it is demonstrated that this simple optical experimental setup is ideal for comparing CS reconstruction algorithms under real measurement conditions, and not just in simulations. Although there are a large number of reconstruction algorithms reported in the literature for use with CS, the algorithms of this work are selected due to their simplicity and known theoretical performance. 

**Figure 3:**
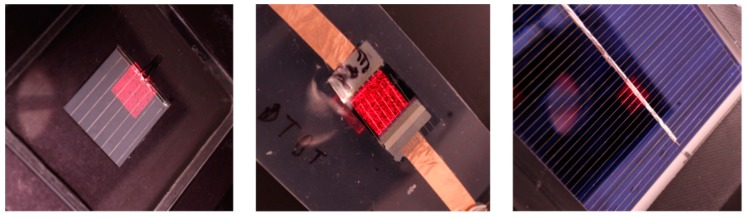
The three samples used in this work, with a random pattern for compressive sampling projected on them. On the left is the c-Si reference cell, in the middle is the organic photovoltaic (OPV) device, and on the right is the large mc-Si cell.

Three samples are used in this work, and an area of 1 cm by 1 cm of each sample is always measured, as well as a monocrystalline silicon (*c*-Si) reference cell, with an active area of 2 cm by 2 cm; an organic photovoltaic (OPV) cell, with an area of 1 cm by 1 cm, non-uniform performance, and a weak current; and a large multicrystalline silicon (*mc*-Si) solar cell with an area of 8 cm by 8 cm, which yields noisy measurements due to its large area. Photocurrent response measurements are acquired at short circuit conditions for all the samples. The samples are presented in Figure 3, with a random pattern projected on them using the DMD optical system. A series of such random patterns are used for compressive sampling.

### 2.3. Sensing Matrix Sparsity

**Figure 4:**
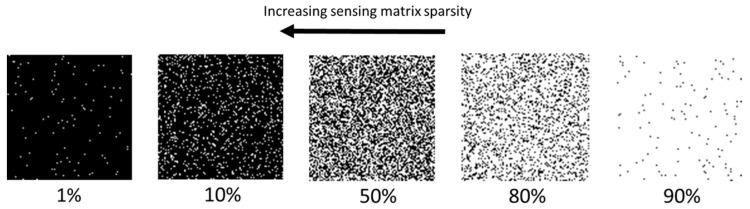
Visualisation of the sparsity of individual patterns of sensing matrices. Sparser patterns have more dark pixels than bright pixels, which is equivalent to a larger proportion of micromirrors being in the “off” state.

The impact of sensing matrix sparsity on the measurement process and on measurement accuracy can be significant. In this work, 100 × 100 pixel random binary sensing matrices are used, with different levels of sparsity, which means that they have a proportion of pixels in the “on” state between 1% and 99%. In this scenario, 50% means that half of the elements of the sensing matrix are in the “on” state, and the rest are in the “off” state. As a result, the projected patterns on the sample have half of their pixels bright (“on”) and the other half dark (“off”). A proportion of 1% simply means that only 1% of the pixels are in the “on” state, resulting in 100 illuminated pixels for a 10,000 pixel projection. As a result, the amplitude of the current response measured, when a series of patterns (rows in a sensing matrix) is projected onto the sample ,will depend on the sparsity of the sensing matrix. This influences signal levels, and so has an impact on the measurement signal-to-noise ratio (SNR). It should be noted that “measurement SNR” is the SNR at the sampling level—the final image SNR of the reconstructed current maps will be lower, and will also depend on the artefacts inserted by the reconstruction procedure. In reality, initial sampling SNR is only one of the factors that influences the final image SNR of the reconstructed image, but it is still a very significant factor for compressive sampling, as will be shown below. Increased sparsity will mean fewer pixels in the “on” state, while reduced sparsity will mean more pixels in the “on” state. Although one could argue that regarding sparsity, 1% and 99% can be the same thing, for the sake of clarity the above convention is adopted throughout this work. This is explained in Figure 4.

It has been demonstrated in CS microscopy that sensing matrix sparsity can have an influence on CS imaging applications [38]. When using very sparse sensing matrices, the probability of having two adjacent pixels in the “on” state at the same time is small. If, in a projected pattern, there are two adjacent pixels in the “on” state simultaneously, the result may be an overlapping excited area in the sample. In CS application cases, as in the optical system of this work, due to light scattering and the diffusion of charge carriers, it may be uncertain to which of the two adjacent pixels the additional measured signal, which contributes to the global current reading of the specific pattern, is generated. Consequently, there may eventually be increased measurement noise in the final reconstructed current map, because of this uncertainty. On the other hand, with very sparse matrices the measured signal is significantly reduced. When using less sparse sensing matrices, many more pixels are in the “on” state, which results in a significant signal amplification, especially when compared with the point-by-point sampling case. The cases when sparser or less-sparse matrices are most appropriate for CS current mapping can eventually depend on the sample to be measured or the background noise of measurements.

## 3. Results

### 3.1. Signal Amplification

**Figure 5:**
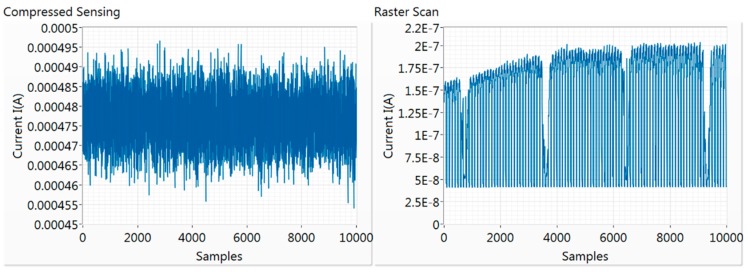
On the left, the current measurements for 10,000 patterns for compressive sampling—all the values are within a very small value range. On the right, the current measurements for a 10,000-pixel point-by-point current map, where the values have a range of 1 order of magnitude.

The photocurrent signal level that a conventional LBIC system has to accurately measure in order to produce the current map can be in the range of nA. In our case, when the optical system is used to implement a point-by-point photocurrent scan, the current values are indeed in the nA range, as can be seen on the right in Figure 5, for the c-Si reference sample. In the same figure, the values of compressive sampling are also presented. All the values are in the range of 0.45–0.50 mA, which means that the current signal is enhanced by at least three orders of magnitude. This is an important feature that can be highly advantageous in cases where the signal level of individual pixel points is very weak to measure with a point-by-point process without a lock-in system. 

All values are within a very narrow range (0.45 mA to 0.50 mA), and this never changes during measurements for a specific sample or a sensing matrix sparsity choice. All the necessary information for reconstructing the current map is within the scatter of the measurements. This means that when acquiring measurements, the minimum and maximum instrument reading range can be set easily in a way that provides a very high dynamic range for the sampling procedure, which can increase accuracy of measurements. This specific feature of compressive sampling is utilised in the next section to correct long-term noise during measurements. Additionally, problematic measurements, such as spikes or zero values, will appear as outliers, and can be excluded easily from the reconstruction process, along with their corresponding pattern. Although the signal levels are greatly enhanced with compressive sampling, actual measurements will still contain noise as any measurement, which always influences the reconstruction process. 

In practice, while signal levels are significantly enhanced by using compressive sampling, the background measurement noise levels are kept relatively stable, depending on the measurement settings of the instrument. In order to show the influence of measurement SNR on the method’s performance, the SNR was calculated for all samples and cases of sensing matrix sparsity. The results are presented in Table 1. The SNR for every projected pattern during compressive sampling is calculated using 30 samples for each measurement (pattern). The measurement SNR is calculated with the Formula (1)

(1) $$\mathrm{SNR}\;(\mathrm{signal}\text{-}\mathrm{to}\text{-}\mathrm{noise}\;\mathrm{ratio})\;=\;\frac{Mean\;Value}{Standard\;Deviation}$$

**Figure 6:**
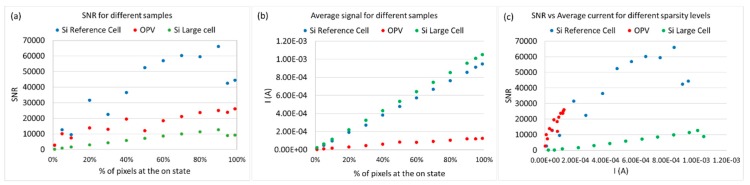
(**a**) Measurement SNR for the three different samples in the case of compressive sampling, with reducing levels of sensing matrix sparsity. (**b**) Signal amplitude (current reading) for the three different samples in the case of compressive sampling, with reducing levels of sensing matrix sparsity. (**c**) SNR as a function of average current of samples, while reducing sparsity levels.

SNR is calculated for each projected pattern, and the measurement SNR is the average for all the patterns. The values of average current and measurement SNR for all three samples, and for different cases of sampling procedures, are presented in Table 1. The difference between compressive sampling and the raster scans (point-by-point scans) regarding signal amplitude and SNR is significant. In particular, for the large area mc-Si cell, the dark current present results in high levels of noise for the point-by-point scan. In all cases of different samples, the signal is amplified at least two orders of magnitude compared to the raster scan, as can be observed in Table 1. The sparsity of sensing matrices also has an effect on SNR of measurements. As it can be observed in Table 1 and in Figure 6a, the SNR increases significantly for all of the samples, with decreasing sparsity levels of sensing matrices. Specific ”falls” of the SNR trend in Figure 6a (for example, at 30% and 95% for the c-Si reference cell) are due to changes in the measurement range for the photocurrent reading of the instrument as the signal increases. This results in slightly higher background noise levels when a higher value is selected for the range of the instrument, when the measured signal reaches the limit for the previous range. For a specific choice of range, the SNR increases steadily, until it saturates before the range changes. This behaviour can be observed from 30% to 90% of sparsity levels for the c-Si reference cell, for 50% to 90% for the OPV, and from 0% to 90% for the large mc-Si cell. This shows that the choice of sensing matrix sparsity for optimising SNR would also depend on the specific instrument used for measurements.

In Figure 6b, the average current measured for each sparsity level is presented. As expected, all the cells demonstrate a linear response, with current increasing while sparsity is decreasing. The silicon devices both demonstrate similar trends, since they have similar efficiencies, while the OPV low-efficiency device produces a much lower current. The falls observed in Figure 6a due to changes in range are not observed in Figure 6b, since what is affected when the range changes is the background noise levels and not the measured signal. The correlation between SNR and measured current is presented in Figure 6c. The same behaviour seen in Figure 6a can be observed for the silicon devices. It is also clear that the OPV device demonstrates the same SNR levels as the c-Si reference device for given measured current values; although the efficiency of the OPV sample is low, and the current gains are not as high as for the silicon devices, its SNR still increases significantly for less sparse sensing matrices.

**Figure 7:**
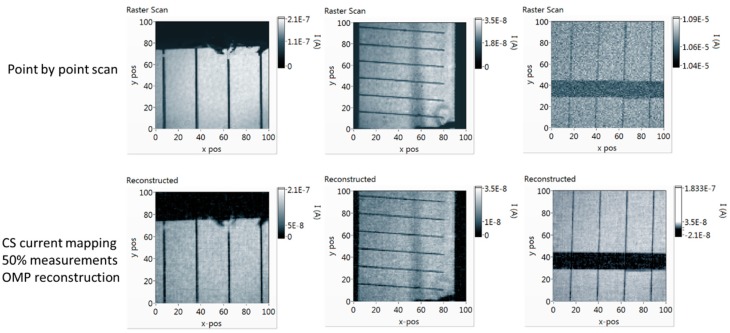
Current maps of the three samples used in this work, with the c-Si reference cell on the left, the OPV in the middle, and the large mc-Si cell on the right. In the top row, point-by-point current maps of the three samples. In the bottom row, CS current maps of the three samples, using the orthogonal matching pursuit (OMP) algorithm for reconstruction and for 50% undersampling.

The influence of measurement SNR can be observed in Figure 7. While a raster scan is possible with this optical system for the two smaller samples, the high noise levels of the large mc-Si sample result in a very noisy current map. On the other hand, with the signal amplification when using compressive sampling, the acquisition of a current map is possible even with such high noise levels. It is clear that the number of pixels in the “on” state of the sensing matrix can be increased in order to amplify the measured signal. This does not affect the reconstruction performance, as will be demonstrated in a following section. Thus, CS current mapping can provide reliable results, even in cases of very weak signals or high noise levels, when a raster scan is not possible. It has to be noted that the SNR analyzed here is the measurement SNR at the sampling stage, and not the final current map SNR. The final current map SNR will also depend on the choice of sensing matrix, transform, reconstruction algorithm, undersampling level, and of course, the initial measurement SNR that is discussed in this work.

### 3.2. Low-Frequency Noise Correction

Although a reference measurement for the laser light source has been implemented into the optical system using a photodiode, there is a more convenient and practical way of removing long-term noise during measurements in the case of compressive sampling. Low frequency noise/drift of signal that is independent of the sample’s instantaneous performance can be due to laser instability or temperature changes of the sample. Such changes can be easily filtered out when compressive sampling is applied. As described in the previous section, when compressive sampling is applied, the complete measurement set spans within a very small range of values. This range is constant for a specific current map and sensing matrix, and any changes due to long-term noise will appear as drifts from this range. In addition, any spikes or other significant instantaneous changes of laser power are visible as outliers, and can be removed from the measurement set without losing any information. This is because fewer measurements than the pixels of the current map are applied, removing one more measurement, and the corresponding pattern will have no effect on the reconstruction.

**Figure 8:**
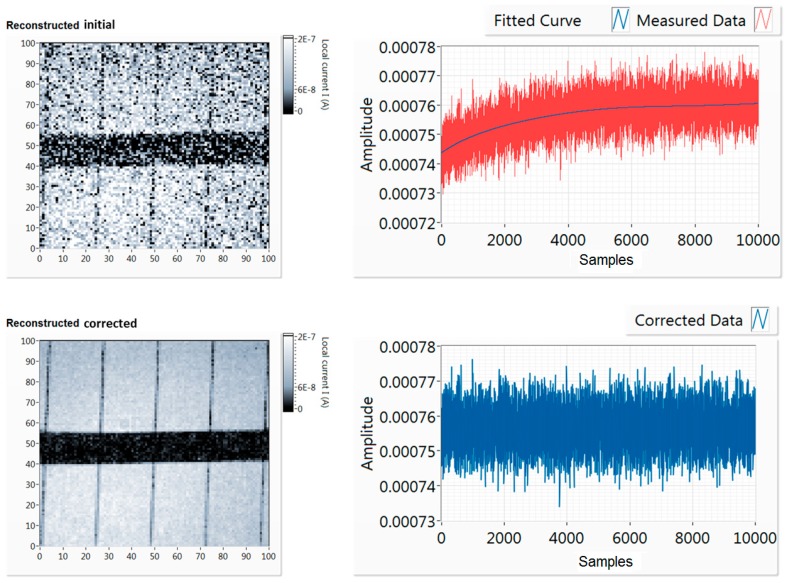
A case of drift correction of sampled data, using 80% pixels “on” patterns. On the top row, the uncorrected map and sampled data, at the bottom, the corrected map and data.

This feature is demonstrated in Figure 8, for a case of compressed sensing current mapping of a small area of the large mc-Si sample with drifting measurement data. The laser source power changed slightly over time, simulating the potential effects of temperature or light source instability. This created a drift of the measured signal, which affected the reconstruction process and resulted in a very noisy current map. The signal was unstable and increased slightly over time. The OMP algorithm is used for reconstruction in this case. As can be observed in Figure 8, this small drift results in a noisy reconstruction of the current map. Nevertheless, since the compressively sampled measurements are expected to always be within a short range of values, this noise can be corrected. Even in this case of more intense deformation of the signal, the actual average signal difference due to this drift is around 2.5%. Still, this affects the reconstruction process if there is no correction of the sampled data. Using a generated curve to normalise the data, the drift is completely removed from the sampled data, after using a polynomial fitting to generate a curve on the corrupted sampled data. Although in most cases it is not necessary, this correction procedure is used for all cases in this work, in order to ensure that any drift of the signal is not affecting reconstruction performance. Such a correction would not be possible with a raster scan, as there would be a chance that real information would be removed.

### 3.3. Reconstruction Performance

For a quantitative evaluation of the performance of the method, depending on sensing matrix sparsity, the point-by-point and reconstructed current maps were compared at a pixel-by-pixel level. This is straightforward to achieve using the DMD optical system, as it includes no moving parts and in both sampling cases, the coordinates of the current maps coincide accurately. Pearson’s correlation coefficient $\rho (\hat{x},x)$ was calculated for different levels of undersampling used for reconstruction, for different levels of sensing matrix sparsity and for the two different algorithms. The correlation coefficient is calculated by dividing the covariance of the point-by-point and reconstructed current map by the product of their standard deviations:

(2) $$\rho (\hat{x},x)=\frac{\mathrm{cov}(\hat{x},x)}{\sigma_{\hat{x}}\cdot \sigma_{x}}$$

where **x** is the point-by-point current map, and $\hat{x}$ is the CS-reconstructed current map, both in vector form. For the case of the large mc-Si sample, a pixel-by-pixel comparison is not possible with this optical system. Due to the large area of this sample, and since no lock-in is used, the raster-scanned current map is very noisy due to high dark current, and cannot be used as a reference for the reconstructed current maps for a pixel-by-pixel comparison.

**Figure 9:**
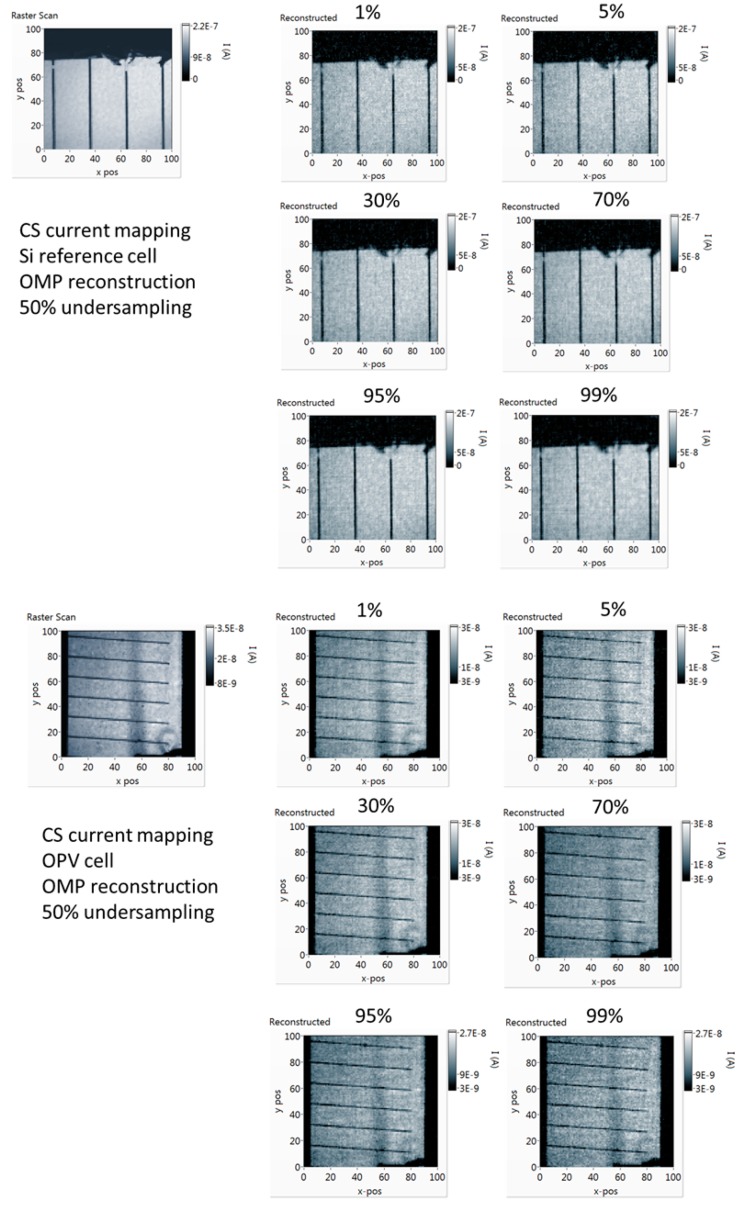
CS current mapping with sensing matrices with different sparsity levels (number of pixels in the “on” state). On the top left, the point-by-point scan is also included for comparison. It can be observed that the differences in reconstruction performance for different sensing matrices are negligible.

In Figure 9, the reconstructed current maps of the c-Si reference cell and the OPV cell are presented along with the raster scan, using the same DMD optical system. By using compressive sampling, the current maps were acquired with half the number of measurements that the raster scan required. A number of sensing matrices with different sparsity levels were used, from 1% of pixels in the “on” state up to 99%. As it can be observed in Figure 9, for this sample, and for a given algorithm, all sensing matrices with different sparsity levels exhibit similar reconstruction performance. In all cases, defects like broken fingers in the silicon device and non-uniformities in the OPV device are clearly imaged. In the case of sensing matrices with 99% of the pixels “on”, it is almost as if the whole sample is illuminated, significantly increasing the measured signal and measurement SNR without affecting the accuracy of the reconstructed current map.

**Figure 10:**
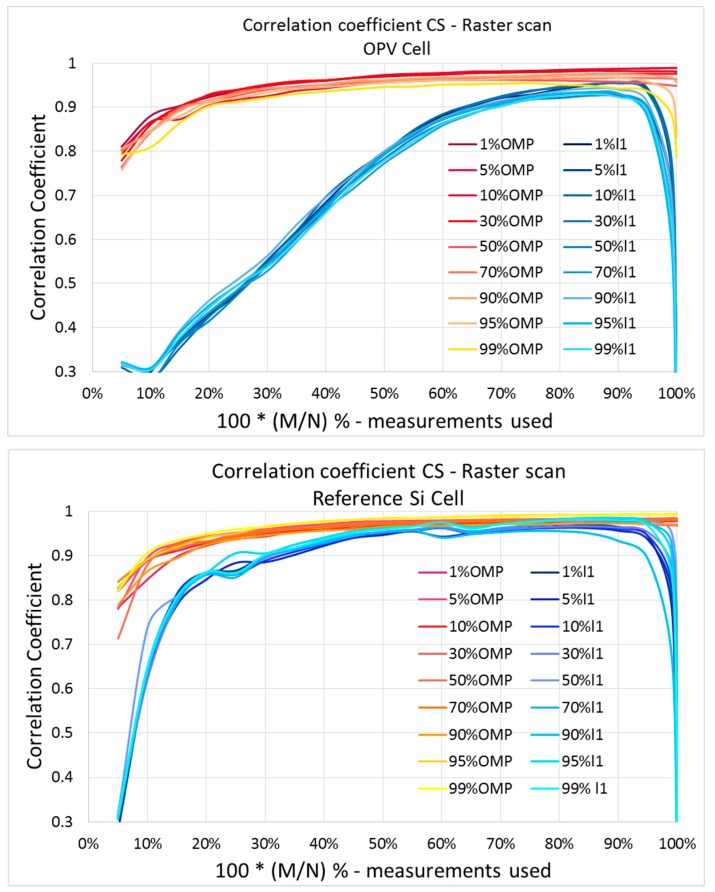
Correlation coefficient as a function of the number of measurements used for reconstruction, for sensing matrices with different sparsity levels, and for the two different algorithms. On the left, the graph for the c-Si reference cell; on the right, the results of the OPV cell.

On the other hand, when using different reconstruction algorithms, the reconstruction performance can vary. In Figure 10, the correlation coefficient between the point-by-point and the CS current maps for the two samples is presented as a function of measurements used for reconstruction, for sensing matrices with different sparsity levels, and for two different reconstruction algorithms (ℓ1, OMP). Although for the c-Si reference cell the differences between different algorithms are not significant, for the OPV sample the reconstruction performance varies significantly between the two algorithms. This shows that some algorithms can have a different performance for different samples, depending on the features of the current map. This has to be taken into consideration when choosing a reconstruction algorithm. Nevertheless, as can be observed in the graphs of Figure 10, sensing matrix sparsity does not affect reconstruction performance for a given reconstruction algorithm. This shows that the right sparsity level of the sensing matrices can be set each time, considering background noise, signal levels, and equipment sensitivity for acquiring the current map of a specific sample.

When approaching 100% of measurements used for reconstruction, the performance of the algorithms, especially that of the ℓ1 algorithm, declines. This is because when measurement noise is included, the optimisation algorithm fails to find a solution for 100% of measurements used, as has been previously demonstrated in [22]. This is because the optimisation algorithm is increasingly constrained as we reach 100%, and has fewer degrees of freedom to filter out noise. There are algorithms available in the literature that can expect some noise in the measurements, and would not have such issues when approaching 100%. For the graphs in Figure 10, reconstruction was implemented for 99.0% as well as 99.9% of sampling, in order to accurately draw these curves. In reality, this area of undersampling is meaningless for compressive sampling, and such problems will not arise in real applications.

## 4. Conclusions

Compressed sensing photocurrent mapping provides fast and reliable measurements with simple experimental layouts. In this work, the signal amplification gains and the ability to optimise CS current mapping by controlling sensing matrix sparsity levels is demonstrated. By setting the right sparsity levels of sensing matrices, a significant increase in the SNR of measurements can be achieved. This provides the means to acquire current maps of samples with very weak currents or high dark currents, where a point-by-point scan would fail. In addition, current mapping systems can be put together without the need of a lock-in amplifier, allowing measurements when the application of lock-in techniques is not possible. The nature of compressive sampling allows long-term noise correction to be applied without the need of a reference measurement of the light source. For this experimental application of CS, the selected sensing matrix sparsity for optimum signal amplification does not affect current map reconstruction performance for a given reconstruction algorithm. As a result, sensing matrix sparsity can be a crucial setting that can be controlled in order to optimise measurement accuracy of CS current mapping.

It is apparent from the results of this work that in CS current mapping, different reconstruction algorithms behave differently for different samples. A future investigation of different algorithms and transforms for this CS application is necessary in order to fully optimise CS current mapping. Since a direct pixel-by-pixel comparison with a raster scan is possible, the DMD-based optical current mapping system of this work offers the opportunity to investigate the performance of different algorithms and transforms for compressive sampling. In this way, tools for this CS application can be evaluated experimentally in a realistic way, including instrument noise and system specific features. Such an evaluation of CS tools can be useful for other optical CS applications, where a comparison with a point-by-point scan is not always possible.

## Figures and Tables

**Table 1:** Average current values and signal-to-noise ratio (SNR) for different samples and sampling methods.

Sampling Method/Pixels in the “on” State		Raster	CS 1%	CS 50%	CS 99%
*Average Current I (A)*	Ref cell	1.37 × 10^−7^	9.57 × 10^−6^	4.77 × 10^−4^	9.48 × 10^−4^
	OPV	2.27 × 10^−8^	1.52 × 10^−6^	8.22 × 10^−5^	1.25 × 10^−4^
	Large cell	1.07 × 10^−5^	2.14 × 10^−5^	5.34 × 10^−4^	1.05 × 10^−3^
*SNR*	Ref cell	54	2637	52,396	44,307
	OPV	19.4	2676	11,963	25,969
	Large cell	1.1	973	7056	9164
